# Anti-Inflammatory Properties of Sirtuin 6 in Human Umbilical Vein Endothelial Cells

**DOI:** 10.1155/2012/597514

**Published:** 2012-10-24

**Authors:** Martha Lappas

**Affiliations:** ^1^Department of Obstetrics and Gynaecology, University of Melbourne, Heidelberg, VIC 3084, Australia; ^2^Mercy Perinatal Research Centre, Mercy Hospital for Women, Heidelberg, VIC 3084, Australia

## Abstract

A prominent feature of inflammatory diseases is endothelial dysfunction. Factors associated with endothelial dysfunction include proinflammatory cytokines, adhesion molecules, and matrix degrading enzymes. At the transcriptional level, they are regulated by the histone deacetylase sirtuin (SIRT) 1 via its actions on the proinflammatory transcription factor nuclear factor-*κ*B (NF-*κ*B). The role of SIRT6, also a histone deacetylase, in regulating inflammation in endothelial cells is not known. The aim of this study was to determine the effect of SIRT6 knockdown on inflammatory markers in human umbilical vein endothelial cells (HUVECs) in the presence of lipopolysaccharide (LPS). LPS decreased expression of SIRT6 in HUVECs. Knockdown of SIRT6 increased the expression of proinflammatory cytokines (IL-1*β*, IL-6, IL-8), COX-prostaglandin system, ECM remodelling enzymes (MMP-2, MMP-9 and PAI-1), the adhesion molecule ICAM-1, and proangiogenic growth factors VEGF and FGF-2; cell migration; cell adhesion to leukocytes. Loss of SIRT6 increased the expression of NF-*κ*B, whereas overexpression of SIRT6 was associated with decreased NF-*κ*B transcriptional activity. Taken together, these results demonstrate that the loss of SIRT6 in endothelial cells is associated with upregulation of genes involved in inflammation, vascular remodelling, and angiogenesis. SIRT6 may be a potential pharmacological target for inflammatory vascular diseases.

## 1. Introduction

Endothelial dysfunction is associated with vasoconstriction, a proinflammatory state, and prothrombotic properties. A number of diseases, including most forms of cardiovascular disease, diabetes, cancer, rheumatoid arthritis, and aging, are associated with endothelial dysfunction [1–3]. Endothelial dysfunction is also a characteristic of bacterial sepsis [4, 5]. To reduce or prevent the high incidence of morbidity and mortality associated with vascular complications, it is essential to understand further the mechanism by which inflammation regulates endothelial function.

Systemic inflammation causes an upregulation of a wide number of factors that lead to severe injury of vascular endothelial cells [6–10]. These include growth factors (basic fibroblast growth factor (bFGF or FGF-2), vascular endothelial growth factor (VEGF)); proinflammatory cytokines (IL-1*β*, IL-6) and chemokines (IL-8); extracellular matrix (ECM) degrading enzymes (plasminogen activator inhibitor-1 (PAI-1), matrix metalloproteinases (MMPs)); cell adhesion molecules (intercellular adhesion molecule (ICAM-1), vascular cell adhesion protein (VCAM)-1, P-selectin); and the cyclooxygenase (COX)-prostaglandin system. Collectively, they participate in the inflammatory response and contribute to a vasoconstrictive and prothrombotic state.

There is now increasing evidence that a number of transcription factors, including the sirtuin (SIRT) family and nuclear factor *κ*B (NF-*κ*B), regulate multiple genes whose products are putatively involved in the regulation of endothelial cell function. The sirtuin family consists of seven enzymes (SIRT1–SIRT7) that share a conserved core catalytic domain but differ in their cellular localisation and tissue distribution [11]. To date, most of the studies have focussed on SIRT1. Numerous genes involved in vascular growth, maturation, and remodelling are dysregulated in SIRT1 deficient endothelial cells [12–15]. Additionally, administration of resveratrol, a SIRT1 activator, improves endothelial dysfunction [16–19] and reduces vascular inflammation in mice [20], two central pathophysiological processes involved in the initiation and progression of cardiovascular disease. Importantly, SIRT1 mediates these effects by interacting and inhibiting the proinflammatory transcription factor NF-*κ*B [16–20].

A recent study in mice has suggested a role for SIRT6 in inflammation [21]; however, the role of SIRT6 in endothelial function is not known. In this study, a human umbilical vein endothelial cell (HUVEC) line will be used to determine the effect of the endotoxin lipopolysaccharide (LPS) on SIRT6 expression. In addition, the effect of SIRT6 inhibition on mediators of vascular inflammation will be determined. Specifically, the effect of SIRT6 siRNA on the expression of proinflammatory cytokines (IL-1*β* and IL-6), COX-prostaglandin system (COX-2 and subsequent prostaglandin release), ECM remodelling enzymes (MMP-2, MMP-9, PAI-1), adhesion molecules (ICAM-1, VCAM-1, P-selectin) and angiogenic growth factors (VEGF and FGF-2) will be assessed. The migration and adhesion properties of SIRT6 deficient HUVECs will also be assessed. In order to elucidate if SIRT6 mediates its effects via NF-*κ*B, luciferase assays will be used to determine the effect of SIRT6 overexpression on NF-*κ*B activity.

## 2. Materials and Methods

### 2.1. Treatment of HUVECs with LPS

HUVECs (HUV-EC-C) were obtained from ATCC (Manassas, VA, USA). Cells were incubated in 25 cm^2^ flask in DMEM/F12 enriched with 10% FCS, 100 U/mL penicillin G, and 100 *μ*g/mL streptomycin until they reached 80% confluence. The cells were the treated with DMEM/F-12 containing 2% heat-inactivated FCS in the absence or presence of 10 *μ*g/mL LPS. After 48 h incubation, cells were collected and assessed for SIRT6 expression by Western blotting as detailed below. Six independent experiments were performed.

### 2.2. Gene Silencing of SIRT6 in HUVECs

HUVECs were incubated in DMEM/F12 enriched with 10% FCS, 100 U/mL penicillin G, and 100 *μ*g/mL streptomycin. Cells were transfected using TransIT-siQUEST reagent according to manufacturer guidelines (Mirus Bio, Madison, WI, USA). Cells were transfected with either 50 nM SIRT6 or nonspecific siRNA from Ambion (Austin, Texas) in DMEM (containing 100 U/mL penicillin G, 100 *μ*g/mL streptomycin) for 72 h. The cells were then treated with either 10 *μ*g/mL LPS (in DMEM/F-12 containing 2% heat-inactivated FCS) for an additional 24 h. Cells were collected and stored at −80°C until assayed for mRNA expression by qRT-PCR and protein expression by Western blotting as detailed below. Media were collected and stored at −80°C until assayed for cytokine and prostaglandin release by ELISA as detailed below. Six independent experiments were performed.

### 2.3. Transient Transfection and Luciferase Assay for Detecting NF-*κ*B Activity

A luciferase assay was utilised to determine possible interactions between SIRT6 and NF-*κ*B. HUVECs were transfected using lipofectamine 2000 as described by the manufacturer (Life Technologies, Carlsbad, CA). Cells were cotransfected with 1 *μ*g reporter (either pNF-*κ*B-luc or negative control; Qiagen) and 1 *μ*g plasmid (empty vector or SIRT6 vector; Origene) for 72 h. The cells were then treated with DMEM/F-12 containing 2% heat-inactivated FCS in the absence or presence of 10 *μ*g/mL LPS for 24 h. The media were collected and assayed for IL-6, IL-8, PGE_2_, and PGF_2*α*_ as detailed above. The cells were harvested in lysis buffer and luminescence activity was measured using the Dual-Glo Luciferase Assay kit (Promega) as instructed. Relative luciferase activity was calculated after normalisation with Renilla activity. The results are expressed as a ratio of luciferase activity of the control group. Three independent experiments were performed.

### 2.4. Enzyme Immunoassays

The release of IL-6 and IL-8 was performed by sandwich ELISA according to the manufacturer's instructions (Invitrogen, Carlsbad, CA). The release of PGE_2_ and PGF_2*α*_ into the incubation medium was assayed using a commercially available competitive enzyme immunoassay kit according to the manufacturer's specifications (Kookaburra Kits from Sapphire Bioscience, NSW, Australia). The human ICAM-1 ELISA kit was purchased from PeproTech, Inc. (Rocky Hill, NJ).

### 2.5. Western Blotting

Assessment of protein expression was analysed by Western blotting as previously described [22], with some minor modifications. Two *μ*g protein was loaded into 10% Mini-PROTEAN TGX Gels (Bio-Rad Laboratories, Hercules, CA). Blots were incubated with 0.5 *μ*g/mL rabbit polyclonal anti-SIRT6 (S4197, Sigma, St. Louis, MO, USA) diluted in blocking buffer (5% skim milk/TBS-T (0.05%)) for 24 h at 4°C. Gels were stripped and reprobed with mouse monoclonal anti-*β*-actin (A5316) for 24 h at 4°C. Membranes were viewed using the Chemi-Doc system (Bio-Rad).

### 2.6. RNA Extraction and Quantitative RT-PCR (qRT-PCR)

Total RNA was extracted using Tri-Reagent according to manufacturer's instructions (Sigma-Aldrich, Saint Louis Missouri). RNA concentrations were quantified using a spectrophotometer (Smart Spec, Bio-Rad). RNA concentration and purity were measured using a NanoDrop ND1000 spectrophotometer (Thermo Scientific, Pittsburgh, PA). RNA was converted to cDNA using the SuperScript VILO cDNA synthesis kit (Invitrogen, Carlsbad, California, USA) according to the manufacturer's instructions. The cDNA was diluted tenfold, and 0.8 *μ*L of cDNA was used to perform RT-PCR using Sensimix Plus SYBR green (Quantace, Alexandria, NSW, Australia) and 100 nM of prevalidated and designed primers (QuantiTect Primer Assays, Qiagen, Germantown, Maryland, USA). The specificity of the product was assessed from melting curve analysis. RNA without reverse transcriptase during cDNA synthesis as well as PCR reactions using water instead of template showed no amplification. Average gene C_T_ values were normalised to the average GAPDH C_T_ values of the same cDNA sample. Fold differences were determined using the comparative C_T_ method, and the results are expressed as a ratio of the average of the control group.

### 2.7. Cell Migration Assay

To assess the migration/invasion ability of SIRT6 deficient cells, a gelatin invasion assay was used. Briefly, transwell inserts (8 *μ*m pore size) were coated on the underside with 0.1% gelatin. HUVECs transfected with NS or SIRT6 siRNA were prepared as detailed above and 5 × 10^5^ cells were allowed to migrate across inserts for 5 hours at 37°C. Unmigrated cells from the upper chamber were removed, and migrated cells in the lower side of the membrane were fixed, stained, and counted. The average number of migrated cells in ten randomly chosen fields of view per insert was taken to quantify the extent of migration. Four independent experiments were performed.

### 2.8. Cell Adhesion Assays

HUVECs transfected with NS or SIRT6 siRNA were prepared as detailed above and 2 × 10^4^ cells in 100 *μ*L per well were added to gelatin precoated 96-well plates and allowed to adhere for 1.5 h at 37°C. Unbound cells were washed, 10 *μ*L of MTT (5 mg/mL stock in PBS) was added per well, and the incubation continued for 4 h. After washing, the bound dye was lysed with 200 *μ*L DMSO and absorbance read at 570 nm. Four independent experiments were performed.

### 2.9. Endothelial-Leukocyte Adhesion Assay

Polymorphonuclear leukocyte (PMN) cells, isolated from whole blood, were a gift from Dr. Katherine Woods (Ludwig Institute for Cancer Research, Austin Hospital, Heidelberg, Victoria, Australia). For these experiments, 48-well plates were used and HUVECs transfected with NS or SIRT6 siRNA were prepared as detailed above. After 3 washings with serum-free medium, 1 × 10^6^ PMN cells per mL were added to each well (final 2.5 × 10^5^ PMN cells per well), and incubated at 37°C for an additional 60 min. After endothelial-leukocyte coculture, nonadherent PMNs cells were removed by washing. The number of adhered PMNs was evaluated by a colorimetric assay using MTT assay as detailed above. Four independent experiments were performed.

### 2.10. Statistical Analysis

Statistical analyses were performed using a commercially available statistical software package (Statgraphics Plus version 3.1, Statistical Graphics Corp., Rockville, Maryland, USA). Two sample comparisons were analysed by a paired sample comparison. For all other comparisons, analysis was performed using a one-way ANOVA (using Tukey HSD correction to discriminate among the means); homogeneity of data was assessed by Bartlett's test, and when significant, data were logarithmically transformed before further analysis. Statistical difference was indicated by a *P* value of less than 0.05. Data are expressed as mean ± standard error of the mean (SEM).

## 3. Results

### 3.1. Effect of LPS on SIRT6 Expression

**Figure 1:**
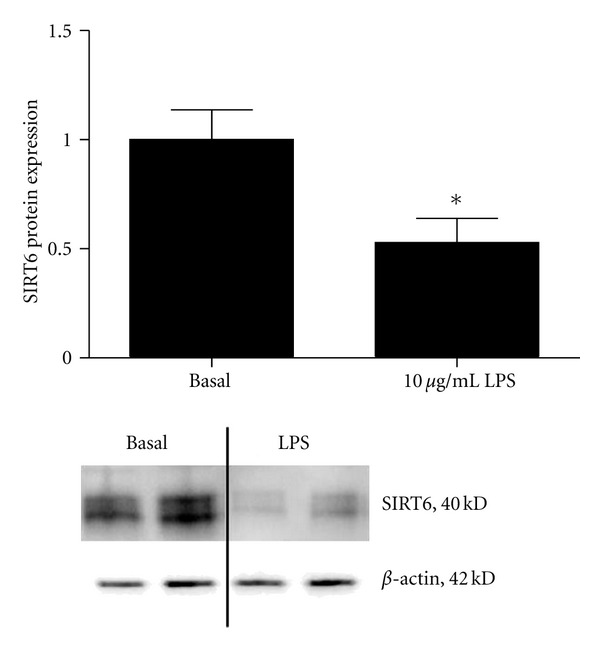
Effect of LPS on SIRT6 expression in HUVECs. HUVECs were incubated in the absence or presence of 10 *μ*g/mL LPS for 48 h. SIRT6 protein was assessed by Western blotting and normalised to *β*-actin. Data is displayed as mean ratio of basal ± SEM (*n* = 6 experiments). *P < 0.05 versus basal (Student's *t*-test). Two representative Western blot images are shown.

The first aim was to determine the effect of LPS on SIRT6 expression in HUVECs. To do this, HUVECs were incubated in the absence or presence of 10 *μ*g/mL LPS for 48 h and protein expression assessed by Western blotting (*n* = 6 experiments). As shown in Figure 1, LPS significantly decreased SIRT6 protein expression, without affecting *β*-actin expression.

### 3.2. Efficiency of SIRT6 Knockdown Using siRNA in HUVECs

**Figure 2:**
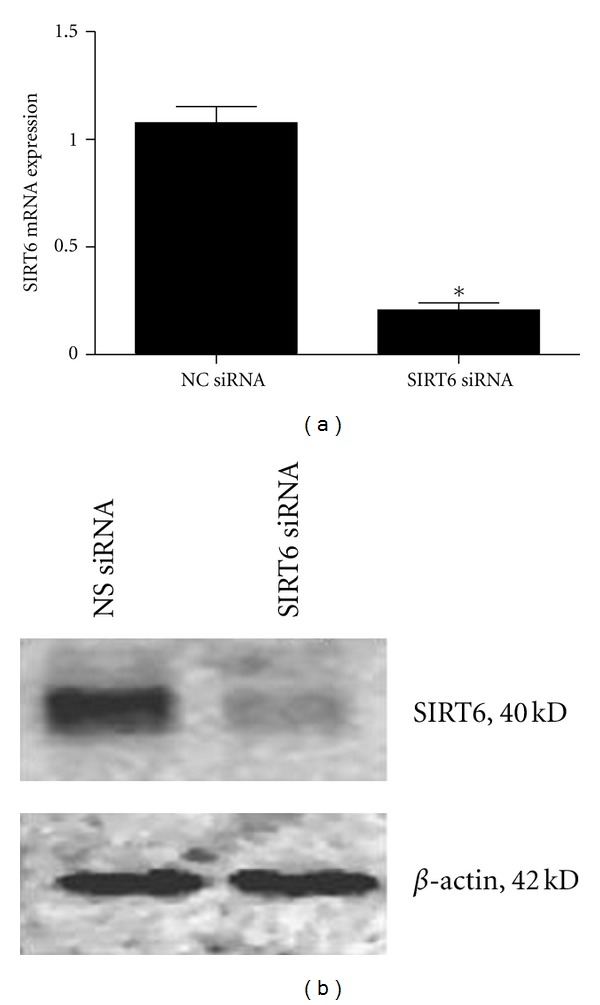
Efficiency of SIRT6 siRNA transfection. HUVECs were transfected with 50 nM SIRT6 or NS siRNA for 96 h (*n* = 6). (a) SIRT6 mRNA expression was quantified by qRT-PCR. SIRT6 expression is displayed as mean ± SEM. *P < 0.05 versus NS siRNA transfected cells. (b) SIRT6 protein was assessed by Western blotting. One representative image is shown.

Having shown that LPS inhibits SIRT6 expression in HUVECs, the next aim was to determine the effect of SIRT6 knockdown on endothelial function in the presence of LPS. To do this, HUVECs were transfected with and without SIRT6 siRNA and nonspecific (NS) siRNA. After 72 h transfection, cells were incubated in the absence or presence of 10 *μ*g/mL LPS for 24 h. The efficacy of transfection was analysed by qRT-PCR and Western blotting. Compared to NS siRNA transfected HUVECs, transfection with SIRT6 siRNA resulted in a significant decrease in SIRT6 mRNA (Figure 2(a)) and protein expression (Figure 2(b)). There was, however, no effect of SIRT6 siRNA on *β*-actin protein expression (Figure 2(b)).

### 3.3. Effect of SIRT6 Knockdown on Cytokine and Chemokine Release and Steady State of Cytokine mRNA

**Figure 3:**
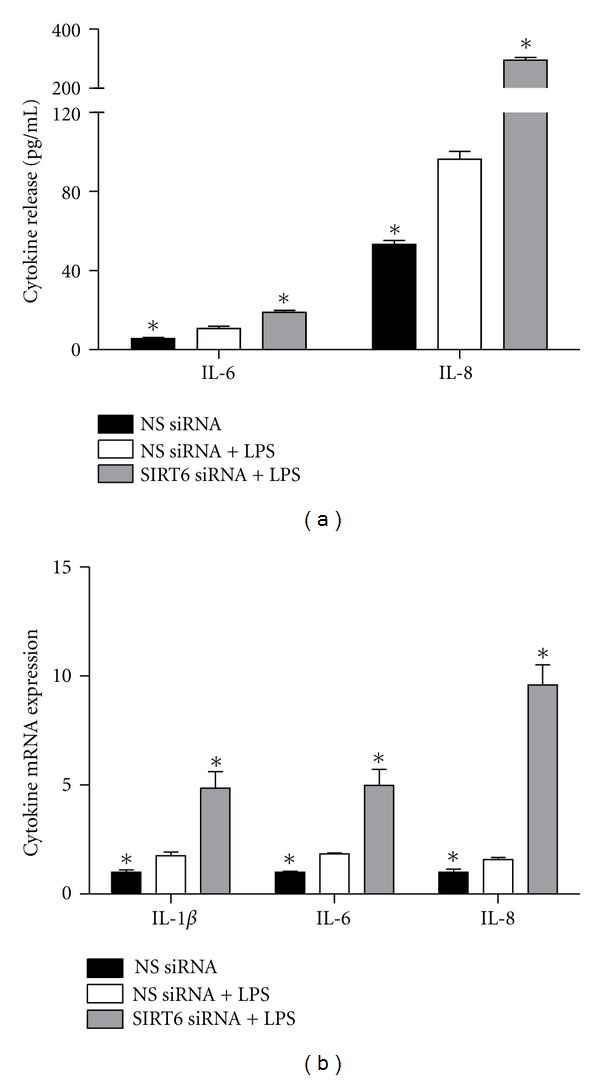
Effect of SIRT6 knockdown on proinflammatory cytokines. HUVECs were transfected with 50 nM SIRT6 or NS siRNA for 72 h followed by 10 *μ*g/mL LPS for 24 h (*n* = 6). (a) IL-6 and IL-8 concentration in the conditioned media was assayed using ELISA. (b) IL-1*β*, IL-6, and IL-8 mRNA expression was quantified by qRT-PCR. Each bar represents the mean ± SEM. *P < 0.05 versus NS siRNA + LPS.

Treatment of HUVECs with LPS significantly increased the release (Figure 3(a)) and gene expression (Figure 3(b)) of IL-1*β*, IL-6, and IL-8. In support with the hypothesis that SIRT6 regulates endothelial cell inflammation, siRNA knockdown of SIRT6 is associated with significantly increased gene expression of the proinflammatory cytokine IL-6 and the chemokine IL-8 (Figure 3(a)). To determine whether inhibition of SIRT6 plays a role in the steady state of cytokine mRNA expression, qRT-PCR analysis was performed after treatment of HUVECs with siRNA. Consistent with the release data, when compared to NS siRNA transfected HUVECs, SIRT6 siRNA induced IL-6 and IL-8 mRNA expression (Figure 3(b)), as well as increasing the mRNA expression of IL-1*β*.

### 3.4. Effect of SIRT6 Knockdown on COX-Prostaglandin Pathway

**Figure 4:**
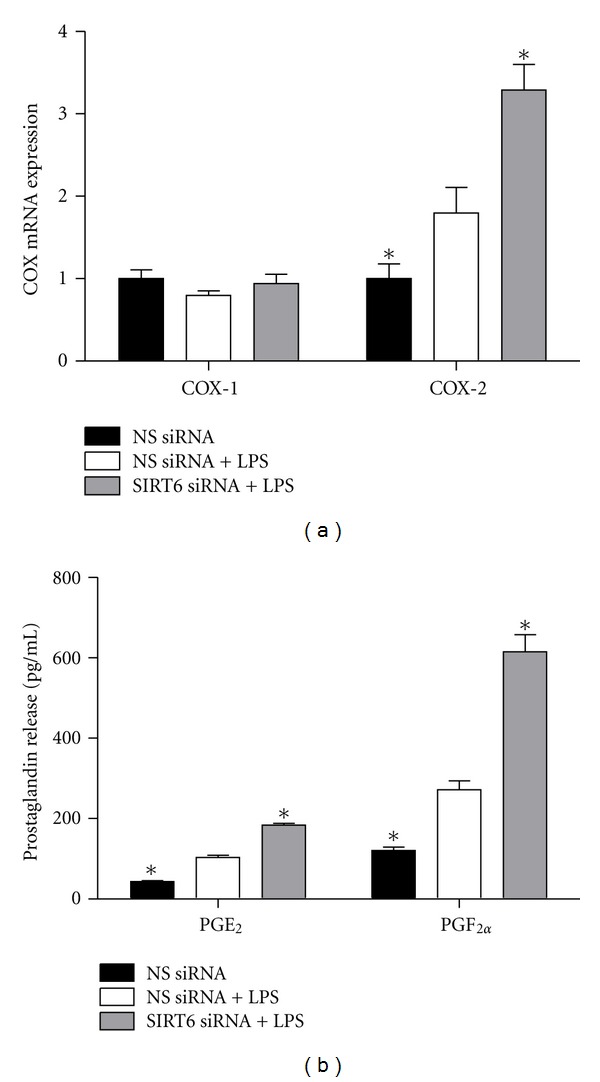
Effect of SIRT6 knockdown on COX-prostaglandin pathway. HUVECs were transfected with 50 nM SIRT6 or NS siRNA for 72 h followed by 10 *μ*g/mL LPS for 24 h (*n* = 6). (a) COX-1 and COX-2 mRNA expression was quantified by qRT-PCR. (b) PGE_2_ and PGF_2*α*_ levels in the conditioned media was assayed using EIA. Each bar represents the mean ± SEM. *P < 0.05 versus NS siRNA + LPS.

The next aim was to determine the effect of SIRT6 siRNA on the COX-prostaglandin system, specifically COX-1 and COX-2 gene expression and subsequent PGE_2_ and PGF_2*α*_ release. LPS significantly induced COX-2 mRNA expression (Figure 4(a)) and release of PGE_2_ and PGF_2*α*_ (Figure 4(b)). As shown in Figure 4, inhibition of SIRT6 significantly increased COX-2 mRNA expression and subsequent prostaglandin release. There was no effect of LPS or SIRT6 knockdown on COX-1 mRNA expression.

### 3.5. Effect of SIRT6 siRNA on ECM Remodelling Enzymes

**Figure 5:**
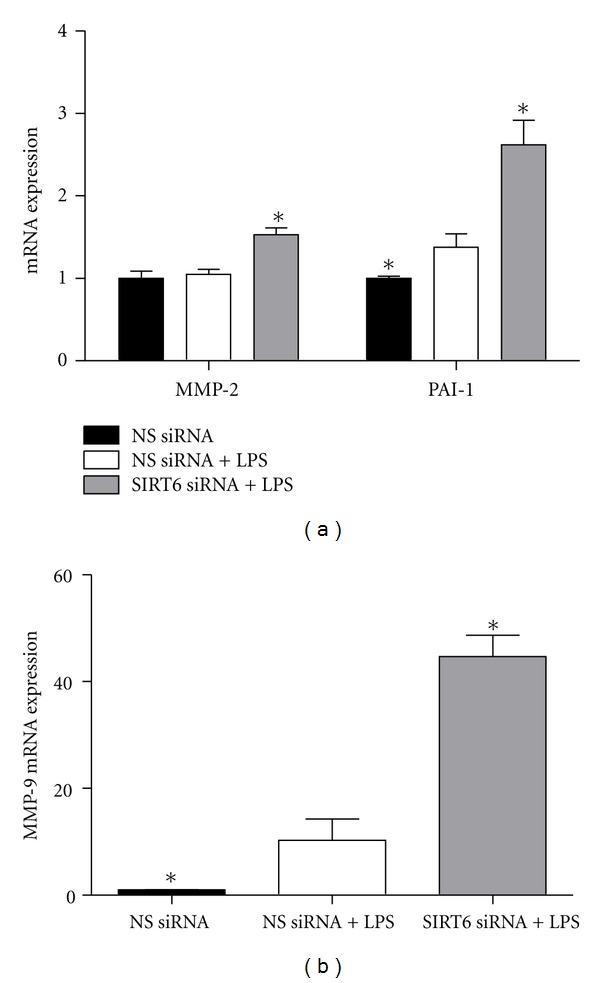
Effect of SIRT6 knockdown on ECM remodelling enzymes. HUVECs were transfected with 50 nM SIRT6 or NS siRNA for 72 h followed by 10 *μ*g/mL LPS for 24 h (*n* = 6). MMP-2, MMP-9 and PAI-1 mRNA expression was quantified by qRT-PCR. Each bar represents the mean ± SEM. *P < 0.05 versus NS siRNA + LPS.

We next sought to determine the effect of SIRT6 knockdown on enzymes involved in ECM degradation and ECM remodelling, namely PAI-1, MMP-2, and MMP-9. As shown in Figure 5, LPS induced MMP-9 and PAI-1 gene expression; an effect that was even higher in SIRT6 deficient HUVECs. On the other hand, there was no effect of LPS or SIRT6 inhibition on MMP-2 gene expression.

### 3.6. Effect of SIRT6 siRNA on Cell Adhesion

**Figure 6:**
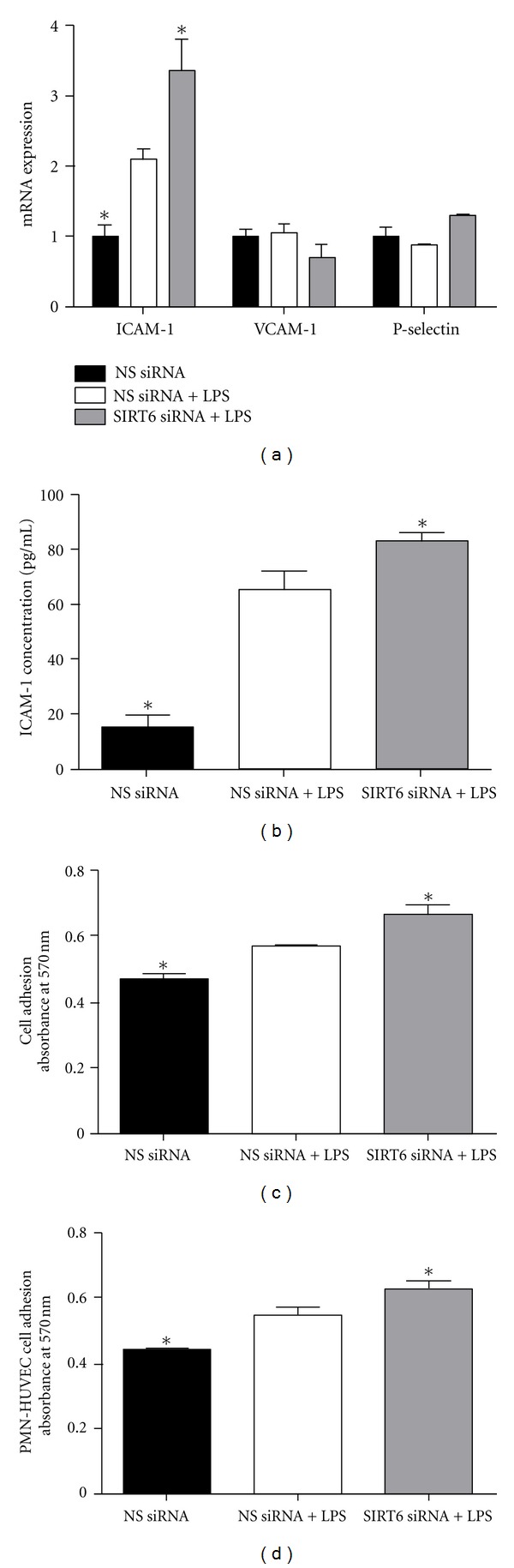
Effect of SIRT6 knockdown on cell adhesion. HUVECs were transfected with 50 nM SIRT6 or NS siRNA for 72 h followed by 10 *μ*g/mL LPS for 24 h (*n* = 6). (a) The gene expression of the adhesion molecules VCAM-1, ICAM-1, and P-selectin was quantified by qRT-PCR. (b) ICAM-1 concentration in the conditioned media was assayed using ELISA. (c) Transfected HUVECs were plated onto gelatin-coated 96-well plates for 1.5 h, and cell adhesion assays were performed using MTT. Extracted dye was quantified by absorbance at 570 nm. Each bar represents the mean ± SEM. *P < 0.05 versus NS siRNA + LPS. (d) PMN cells were added to each well and were incubated at 37°C for 60 min. The number of adhered PMNs was evaluated by a colorimetric assay using MTT assay and quantification by absorbance at 570 nm. Each bar represents the mean ± SEM. *P < 0.05 versus NS siRNA + LPS.

To investigate putative promigratory effects of SIRT6 siRNA, endothelial VCAM-1, ICAM-1, and P-selectin mRNA expression was quantified by qRT-PCR. LPS significantly increased the gene expression of the cell adhesion molecule ICAM-1 (Figure 6(a)). This was also associated with an increase in the secretion of ICAM-1 into the conditioned media (Figure 6(b)). Additionally, when compared to LPS alone, inhibition of SIRT6 significantly increased ICAM-1 gene expression and release (Figures 6(a), 6(b)). There was, however, no effect of LPS or LPS plus SIRT6 siRNA on VCAM-1 and P-selectin gene expression (Figure 6(a)).

To evaluate the role of SIRT6 in the cell adhesion potential of HUVECs, two different assays were performed. In the first assay, NS or SIRT6 siRNA transfected HUVECs were plated in 1% gelatin-coated 96 well plates for 1.5 h. Thereafter, unattached cells were washed and MTT colorimetric assay was used to determine the number of adherent cells. The optical density of the resulting solution was determined at 570 nm. As depicted in Figure 6(c), in NS siRNA transfected cells, LPS induced an increase in absorbance, indicating an increase in cell adhesion. This increase in cell adhesion was further augmented in SIRT6 deficient HUVECs.

The effect of SIRT6 siRNA transfected HUVECs to bind to leukocytes was also assessed. In these experiments, leukocytes were added to confluent monolayers of HUVECs transfected with NS or SIRT6 siRNA. As shown in Figure 6(d), in NS siRNA transfected HUVECs, stimulation with LPS induced a significant increase in the adhesion of leukocytes. When SIRT6 was knocked out in these cells, the adhesion of leukocytes was further increased.

### 3.7. Effect of SIRT6 siRNA on Cell Migration and Angiogenesis

**Figure 7:**
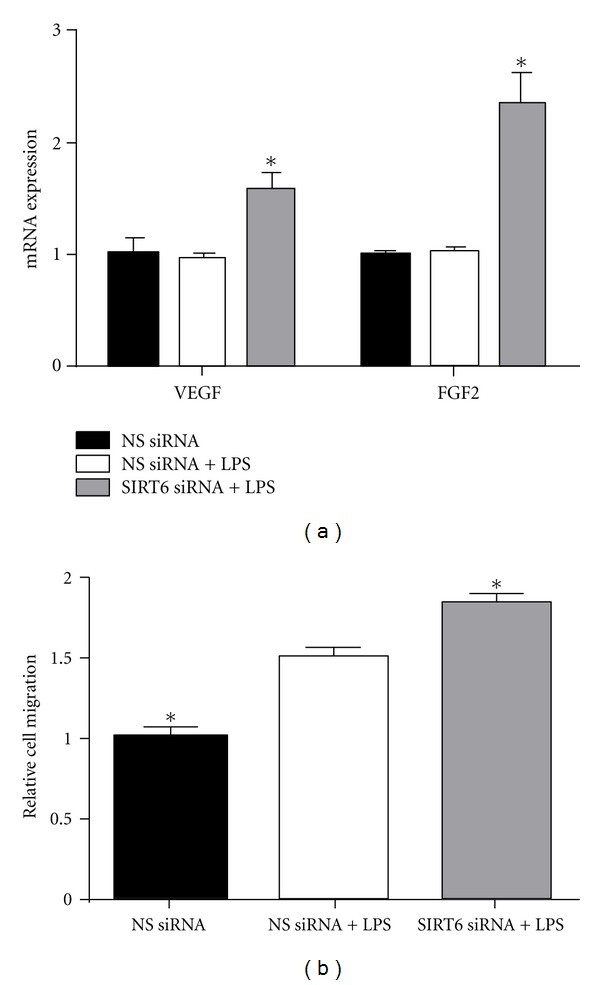
Effect of SIRT6 siRNA on HUVEC angiogenic proteins and cell migration. HUVECs were transfected with 50 nM SIRT6 or NS siRNA for 72 h followed by 10 *μ*g/mL LPS for 24 h (*n* = 4). (a) The gene expression of the proangiogenic proteins VEGF and FGF-2 was quantified by qRT-PCR. Each bar represents the mean ± SEM. *P < 0.05 versus NS siRNA + LPS. (b) Transfected HUVECs were put into transwell inserts coated on the underside with 0.1% gelatin. The total number of migrating cells were counted after 5 h at 37°C. Fold change was calculated relative to NS siRNA transfected cells. Each bar represents the mean ± SEM. *P < 0.05 versus NS siRNA + LPS.

Although there was no effect of LPS on the expression of the angiogenic growth factors VEGF and FGF2 (Figure 7(a)), in HUVECs transfected with SIRT6 siRNA, VEGF and FGF2 gene expression was significantly upregulated (Figure 7(b)). To measure the migration potential of siRNA-treated HUVECs, an invasion chamber assay was performed. This assay measures the number of cells migrating through pores of a polycarbonate filter, simulating a basement membrane. 72 h after transfection, the number of cells on the underside of each membrane was counted and the mean calculated. The effect of SIRT6 knockdown on HUVEC cell migration is depicted in Figure 7(c). In NS siRNA transfected cells, LPS treatment increased the mean number of cells that migrated through the membrane. When compared to LPS treated NS siRNA transfected HUVECs, the mean number of cells that migrated through the membrane was increased in SIRT6 siRNA transfected HUVECs. 

### 3.8. SIRT6 Regulates the Expression and Activity of NF-*κ*B

**Figure 8:**
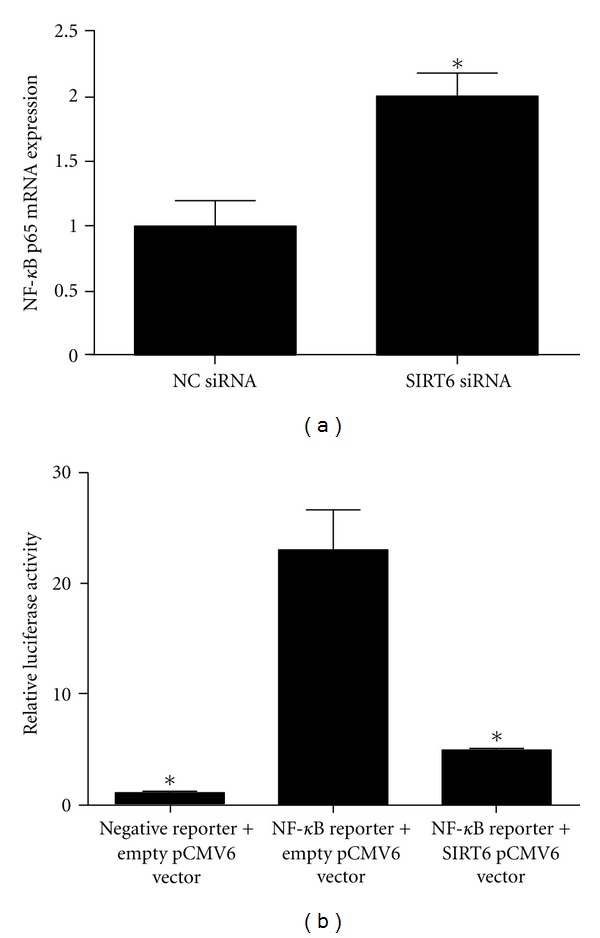
SIRT6 regulates inflammation via NF-*κ*B. (a) HUVECs were transfected with 50 nM SIRT6 or NS siRNA for 96 h (*n* = 6). NF-*κ*B p65 mRNA expression was quantified by qRT-PCR. Each bar represents the mean ± SEM. *P < 0.05 versus NS siRNA transfected cells. (b) HUVECs were cotransfected with 1 *μ*g negative control construct and NF-*κ*B reporter construct, along with 1 *μ*g empty or SIRT6 pCMV6 vector for 72 h. Promoter activity is expressed as a ratio of luciferase activity of the control group, which was set as 1. Each bar represents the mean ± SEM (*n* = 3 experiments). *P < 0.05 versus cells transfected with NF-*κ*B reporter plus empty pCMV6 vector.

The final aim of this study was to determine if SIRT6 regulates proinflammatory factors in HUVECs via the NF-*κ*B pathway. To assess this, the effect of SIRT6 siRNA on NF-*κ*B transcription was investigated first. As shown in Figure 8(a), knockdown of SIRT6 significantly increased NF-*κ*B p65 mRNA expression. Next, whether SIRT6 regulates the transcriptional activity of NF-*κ*B by luciferase assays using NF-*κ*B-luc reporter was determined. The cells were also transfected with SIRT6 expression vector or control vector pcDNA3.1. As shown in Figure 8(b), transfection with NF-*κ*B luc reporter significantly increased luciferase activity. However, overexpression of SIRT6 inhibited NF-*κ*B-mediated luciferase reporter expression in HUVECs.

**Figure 9:**
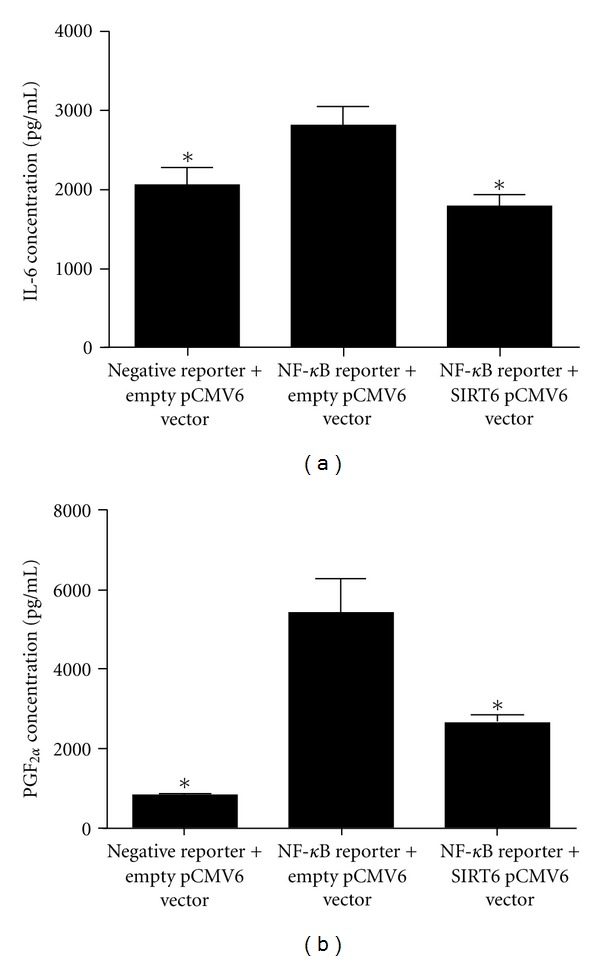
SIRT6 regulates proinflammatory mediators via NF-*κ*B. HUVECs were cotransfected with 1 *μ*g negative control construct and NF-*κ*B reporter construct, along with 1 *μ*g empty or SIRT6 pCMV6 vector for 72 h. (a) IL-6 and (b) PGF_2*α*_, concentration in condition medium. Each bar represents the mean ± SEM (*n* = 3 experiments). *P < 0.05 versus cells transfected with NF-*κ*B reporter plus empty pCMV6 vector.

The effect of NF-*κ*B overexpression with and without co-transfection with SIRT6 in HUVECs on a number of NF-*κ*B target genes was also evaluated. As shown in Figure 9, overexpression of NF-*κ*B significantly increased IL-6 and PGF_2*α*_ levels in the conditioned media. This increase was inhibited in HUVECs that also overexpressed SIRT6. Similar results were obtained for IL-8 and PGE_2_ (data not shown).

## 4. Discussion

Epidemiological, clinical, and animal studies demonstrate that bacterial infection can induce endothelial dysfunction cells [6–10, 23]. Recent reports highlight an important protective role for SIRT1 in vascular dysfunction. For example, infection decreases SIRT1 expression and activity [22, 24] and activation of SIRT1 exerts beneficial effects on endothelial cell dysfunction [18, 25]. This is the first study to report that LPS can also decrease SIRT6 expression in HUVECs. This led to the hypothesis that SIRT6 may also play an important role in infection induced endothelial dysfunction. In order to test this theory, SIRT6 was inhibited in HUVECs using siRNA, and then a number of factors known to play an important role in vascular disease were investigated. In SIRT6 deficient cells, in the presence of LPS, there was an increase in the expression of the proinflammatory cytokines IL-1*β* and IL-6, the chemokine IL-8, ECM degrading enzymes MMP-9 and PAI-1, COX-prostaglandin system, and the angiogenic growth factors FGF-2 and VEGF. The loss of SIRT6 in HUVEC was associated with increased expression of the cell adhesion molecule ICAM-1, as well as increased cell adhesion properties. In addition, loss of SIRT6 increases the expression of the proinflammatory transcription factor NF-*κ*B, whereas overexpression of SIRT6 decreases the transcriptional activity of NF-*κ*B as well as the expression of its target genes. Collectively, this suggests that SIRT6 is a negative regulator of vascular inflammation. Thus, activation of SIRT6 may be beneficial for many inflammatory diseases associated with endothelial dysfunction.

Endothelial cells themselves are a major source of proinflammatory cytokines, which are significantly enhanced during inflammation [9]. These proinflammatory stimuli can induce a phenotypic change of the quiescent endothelium to an activated endothelium. This results in an increase or induction of the endothelial expression of adhesion molecules and chemokines (chemoattractant cytokines), leading to leukocyte recruitment, adhesion, and infiltration into the subendothelium. Activated endothelium also leads to an increase in cytokine expression and production that may act in an autocrine fashion to enhance endothelial cell activation further. In this study, LPS induced the expression and release of the proinflammatory cytokines IL-1*β* and IL-6, the chemokine IL-8; this increase was even higher in SIRT6 deficient HUVECs. As expected, LPS stimulation induced the adhesion molecule ICAM-1 as well as leukocyte cell adhesion to HUVECs, and this increase was further augmented in SIRT6 deficient cells. Of note, there was no effect of LPS or SIRT6 inhibition on the expression of VCAM-1 or P-selection. Although there are no studies on SIRT6 and adhesion molecules, these studies are in support of the few other studies demonstrating that SIRT6 regulates inflammatory cytokines [26, 27].

In the process of vascular inflammation, activated endothelial cells can also migrate to surrounding tissues to spur the formation of new blood vessels, or angiogenesis, which plays an important role in a number of pathologies such as atherosclerosis, diabetes, and tumour development [28–30]. Indeed, in experimental studies, LPS has been shown to promote human endothelial cell migration. In this study, LPS stimulation not only promoted HUVEC migration but the effect was intensified in SIRT6 deficient HUVECs. VEGF and FGF-2 are two angiogenic growth factors whose activation leads to dysregulated blood vessel formation as seen in various chronic inflammatory conditions such as cancer and rheumatoid arthritis [28, 29]. In this study, SIRT6 deficient HUVECs had increased levels of both VEGF and FGF-2.

COX-2 and its proinflammatory products have been implicated in the pathogenesis of several inflammatory diseases including diabetes and atherosclerosis [31]. In addition, COX-2 can also contribute to the pathogenesis of endothelial dysfunction by disrupting vascular homeostasis. It also generates vasoconstrictor substances, including vasoconstrictor prostanoids that have proatherogenic effects [32, 33]. This study demonstrates that in the presence of infection, SIRT6 is a regulator of the COX-prostaglandin pathway in endothelial cells. Specifically, the loss of SIRT6 in HUVECs resulted in a significant increase in COX-2 expression concomitant with an increase in PGE_2_ and PGF_2*α*_ release.

MMPs play a key role in the processes of remodelling and reorganisation of the vasculature, which is a key feature of endothelial dysfunction [34, 35]. PAI-1, a member of the serine protease inhibitor family, is a key inhibitor of fibrinolysis; impaired endothelial fibrinolytic potential contributes to the development of thrombotic attacks and atherosclerosis [36, 37]. In addition, PAI-1 is also involved degradation of the ECM [36, 37]. In this study, siRNA knockdown of SIRT6 in HUVECs increased PAI-1 and MMP-9 expression. This is the first study to demonstrate a role for SIRT6 in the regulation of matrix proteins in endothelial cells; however, a role for SIRT1 has been reported [38].

NF-*κ*B is a master regulatory component driving many inflammatory diseases; it is thought to play an important role in endothelial cell function [1, 39, 40]. There is now much evidence to show that SIRT1 exerts many of its protective effects in endothelial cells by inhibiting NF-*κ*B activity [41]. In this study, knockdown of SIRT6 was associated with an increase in NF-*κ*B expression. Additionally, using luciferase assays, NF-*κ*B transcriptional activity was significantly reduced in HUVECs that also overexpressed SIRT6. This decrease was also associated with a decrease in the expression of NF-*κ*B target genes, namely, IL-6 and PGF_2*α*_. Collectively, this suggests that in the presence of SIRT6, HUVECs display hypoactive NF-*κ*B, leading to decreased transcription of proinflammatory genes. Our data is in support of recent studies that report increased expression of NF-*κ*B and its target genes in a number of tissues of SIRT6deficient mice [21].

## 5. Conclusions

Functional alterations of the endothelium are hallmark features of many inflammatory diseases, including diabetes, hypertension, atherosclerosis, rheumatoid arthritis, and aging [1, 42–44]. Therefore, the inhibition of inflammatory mediators in activated endothelial cells might be of great benefit in the maintenance of endothelial homeostasis and the prevention of vascular diseases. This study highlights the relevance of SIRT6 as an important player in inflammatory reactions of the human endothelium in the context of sepsis. There is now growing literature implicating gut bacteria on the development of insulin resistance [45], cardiovascular disease [46], and atherosclerosis [47]. It is, therefore, tempting to speculate that activators of SIRT6 might be an effective strategy for a number of inflammatory vascular diseases such as diabetes and atherosclerosis.

## References

[1] Csiszar A, Wang M, Lakatta EG, Ungvari Z (2008). Inflammation and endothelial dysfunction during aging: role of NF-*κ*B. *Journal of Applied Physiology*.

[2] Cai H, Harrison DG (2000). Endothelial dysfunction in cardiovascular diseases: the role of oxidant stress. *Circulation Research*.

[3] De Vriese AS, Verbeuren TJ, Van De Voorde J, Lameire NH, Vanhoutte PM (2000). Endothelial dysfunction in diabetes. *British Journal of Pharmacology*.

[4] Szabó C, Cuzzocrea S, Zingarelli B, O’Connor M, Salzman AL (1997). Endothelial dysfunction in a rat model of endotoxic shock: importance of the activation of poly (ADP-ribose) synthetase by peroxynitrite. *Journal of Clinical Investigation*.

[5] Schouten M, Wiersinga WJ, Levi M, Van Der Poll T (2008). Inflammation, endothelium, and coagulation in sepsis. *Journal of Leukocyte Biology*.

[6] Kuwano T, Nakao S, Yamamoto H (2004). Cyclooxygenase 2 is a key enzyme for inflammatory cytokine-induced angiogenesis. *FASEB Journal*.

[7] Szekanecz Z, Koch AE (2007). Mechanisms of disease: angiogenesis in inflammatory diseases. *Nature Clinical Practice Rheumatology*.

[8] Sprague AH, Khalil RA (2009). Inflammatory cytokines in vascular dysfunction and vascular disease. *Biochemical Pharmacology*.

[9] Tedgui A, Mallat Z (2006). Cytokines in atherosclerosis: pathogenic and regulatory pathways. *Physiological Reviews*.

[10] Galkina E, Ley K (2007). Vascular adhesion molecules in atherosclerosis. *Arteriosclerosis, Thrombosis, and Vascular Biology*.

[11] Michishita E, Park JY, Burneskis JM, Barrett JC, Horikawa I (2005). Evolutionarily conserved and nonconserved cellular localizations and functions of human SIRT proteins. *Molecular Biology of the Cell*.

[12] Potente M, Ghaeni L, Baldessari D (2007). SIRT1 controls endothelial angiogenic functions during vascular growth. *Genes and Development*.

[13] Stein S, Matter CM (2011). Protective roles of SIRT1 in atherosclerosis. *Cell Cycle*.

[14] Kaur G, Roberti M, Raul F, Pendurthi UR (2007). Suppression of human monocyte tissue factor induction by red wine phenolics and synthetic derivatives of resveratrol. *Thrombosis Research*.

[15] Csiszar A, Labinskyy N, Pinto JT (2009). Resveratrol induces mitochondrial biogenesis in endothelial cells. *American Journal of Physiology*.

[16] Do GM, Kwon EY, Kim HJ (2008). Long-term effects of resveratrol supplementation on suppression of atherogenic lesion formation and cholesterol synthesis in apo E-deficient mice. *Biochemical and Biophysical Research Communications*.

[17] Das S, Alagappan VKT, Bagchi D, Sharma HS, Maulik N, Das DK (2005). Coordinated induction of iNOS-VEGF-KDR-eNOS after resveratrol consumption: a potential mechanism for resveratrol preconditioning of the heart. *Vascular Pharmacology*.

[18] Zhang QJ, Wang Z, Chen HZ (2008). Endothelium-specific overexpression of class III deacetylase SIRT1 decreases atherosclerosis in apolipoprotein E-deficient mice. *Cardiovascular Research*.

[19] Baur JA, Sinclair DA (2006). Therapeutic potential of resveratrol: the in vivo evidence. *Nature Reviews Drug Discovery*.

[20] Pearson KJ, Baur JA, Lewis KN (2008). Resveratrol delays age-related deterioration and mimics transcriptional aspects of dietary restriction without extending life span. *Cell Metabolism*.

[21] Kawahara TLA, Michishita E, Adler AS (2009). SIRT6 links histone H3 lysine 9 deacetylation to NF-*κ*B-dependent gene expression and organismal life span. *Cell*.

[22] Lappas M, Mitton A, Lim R, Barker G, Riley C, Permezel M (2011). SIRT1 is a novel regulator of key pathways of human labor. *Biology of Reproduction*.

[23] Libby P, Ridker PM, Maseri A (2002). Inflammation and atherosclerosis. *Circulation*.

[24] Shen Z, Ajmo JM, Rogers CQ (2009). Role of SIRT1 in regulation of LPS- or two ethanol metabolites-induced TNF-*α* production in cultured macrophage cell lines. *American Journal of Physiology*.

[25] Yuan Q, Chen L, Xiang DX, Li YJ, Hu CP (2011). Effect of resveratrol derivative BTM-0512 on high glucose-induced dysfunction of endothelial cells: role of SIRT1. *Canadian Journal of Physiology and Pharmacology*.

[26] Van Gool F, Gallí M, Gueydan C (2009). Intracellular NAD levels regulate tumor necrosis factor protein synthesis in a sirtuin-dependent manner. *Nature Medicine*.

[27] Minagawa S, Araya J, Numata T (2011). Accelerated epithelial cell senescence in IPF and the inhibitory role of SIRT6 in TGF-*β*-induced senescence of human bronchial epithelial cells. *American Journal of Physiology*.

[28] Carmeliet P, Jain RK (2000). Angiogenesis in cancer and other diseases. *Nature*.

[29] Carmeliet P (2005). Angiogenesis in life, disease and medicine. *Nature*.

[30] Griffioen AW, Molema G (2000). Angiogenesis: potentials for pharmacologic intervention in the treatment of cancer, cardiovascular diseases, and chronic inflammation. *Pharmacological Reviews*.

[31] Natarajan R, Nadler JL (2004). Lipid inflammatory mediators in diabetic vascular disease. *Arteriosclerosis, Thrombosis, and Vascular Biology*.

[32] Feletou M, Huang Y, Vanhoutte PM (2011). Endothelium-mediated control of vascular tone: COX-1 and COX-2 products. *British Journal of Pharmacology*.

[33] Davidge ST (2001). Prostaglandin H synthase and vascular function. *Circulation Research*.

[34] Galis ZS, Khatri JJ (2002). Matrix metalloproteinases in vascular remodeling and atherogenesis: the good, the bad, and the ugly. *Circulation Research*.

[35] Sluijter JPG, De Kleijn DPV, Pasterkamp G (2006). Vascular remodeling and protease inhibition—bench to bedside. *Cardiovascular Research*.

[36] Lijnen HR (2005). Pleiotropic functions of plasminogen activator inhibitor-1. *Journal of Thrombosis and Haemostasis*.

[37] Stefansson S, McMahon GA, Petitclerc E, Lawrence DA (2003). Plasminogen activator inhibitor-1 in tumor growth, angiogenesis and vascular remodeling. *Current Pharmaceutical Design*.

[38] Li L, Zhang HN, Chen HZ (2011). SIRT1 acts as a modulator of neointima formation following vascular injury in mice. *Circulation Research*.

[39] Li XC, Zhuo JL (2008). Nuclear factor-kappaB as a hormonal intracellular signaling molecule: focus on angiotensin II-induced cardiovascular and renal injury. *Current Opinion in Nephrology and Hypertension*.

[40] Liang CJ, Wang SH, Chen YH (2011). Viscolin reduces VCAM-1 expression in TNF-*α*-treated endothelial cells via the JNK/NF-*κ*B and ROS pathway. *Free Radical Biology and Medicine*.

[41] Breitenstein A, Stein S, Holy EW (2011). Sirt1 inhibition promotes in vivo arterial thrombosis and tissue factor expression in stimulated cells. *Cardiovascular Research*.

[42] Van Gaal LF, Mertens IL, De Block CE (2006). Mechanisms linking obesity with cardiovascular disease. *Nature*.

[43] Kim JA, Montagnani M, Kwang KK, Quon MJ (2006). Reciprocal relationships between insulin resistance and endothelial dysfunction: molecular and pathophysiological mechanisms. *Circulation*.

[44] Kerekes G, Soltesz P, Nurmohamed MT (2012). Validated methods for assessment of subclinical atherosclerosis in rheumatology. * Nature Reviews Rheumatolog*.

[45] Caricilli AM, Picardi PK, de Abreu LL (2011). Gut microbiota is a key modulator of insulin resistance in TLR 2 knockout mice. *PLOS Biology*.

[46] Wang Z, Klipfell E, Bennett BJ (2011). Gut flora metabolism of phosphatidylcholine promotes cardiovascular disease. *Nature*.

[47] Koren O, Spor A, Felin J (2011). Human oral, gut, and plaque microbiota in patients with atherosclerosis. *Proceedings of the National Academy of Sciences of the United States of America*.

